# New Model of Integrated Care for Patients with Immune-Mediated Inflammatory Diseases

**DOI:** 10.5334/ijic.7741

**Published:** 2025-06-11

**Authors:** Esther Chamorro-de-Vega, C. M. González, L. Menchén, O. Baniandrés, A. Herranz, C. Lobo-Rodríguez, R. Romero-Jiménez, A. Ais-Larisgoitia, E. Lobato-Matilla, A. López-Esteban, A. López-Calleja, I. Marín-Jiménez, I. Monteagudo, P. Morales de Los Ríos, J. C. Nieto, M. Ferris-Villanueva, M. J. Lizcano, M. P. Simón Moreno, M. Sanjurjo, S. García de Sanjosé

**Affiliations:** 1 Pharmacy Department-CEIMI, Hospital General Universitario Gregorio Marañón, Madrid, Spain; 2 Instituto de Investigación Sanitaria Gregorio Marañón, Madrid, Spain; 3 Rheumatology Department-CEIMI, Hospital General Universitario Gregorio Marañón, Madrid, Spain; 4 Gastroenterology Department-CEIMI, Hospital General Universitario Gregorio Marañón, Madrid, Spain; 5 Dermatology Department-CEIMI, Hospital General Universitario Gregorio Marañón, Madrid, Spain; 6 Nursing Group-CEIMI, Hospital General Universitario Gregorio Marañón, Madrid, Spain; 7 Admission and clinical documentation service-CEIMI, Hospital General Universitario Gregorio Marañón, Madrid, Spain; 8 Hospital Hospital Medical Directorate, Hospital General Universitario Gregorio Marañón, Madrid, Spain

**Keywords:** integrative medicine, health care reform, comprehensive health care, patient-centered care, autoimmune diseases, patient care team, health-care quality

## Abstract

**Introduction:**

The complexity and transversality of the care for patients with immune-mediated inflammatory diseases (IMIDs) represents a challenge for the usual structure of health care services and requires a transformation of existing clinical management models. We describe the design, implementation and evaluation of a new collaborative care model for patients with IMIDs.

**Description:**

A group of multidisciplinary professionals including specialists from the rheumatology, gastroenterology, dermatology and pharmacy services designed and implemented an innovative health care model for patients with IMIDs that has changed the traditional model of care. One of the main challenges is the transversal leadership system in collaboration with all the hospital services involved, including the Medical Directorate of the hospital and patients, which promotes greater responsibility and empowerment.

**Discussion:**

Although IMID patient care by a multidisciplinary team is widely recommended, guidelines for IMID-specific models are lacking and few authors have reported controversial results of this strategy.

**Conclusions:**

CEIMI is a pioneering collaborative and multidisciplinary care model for patients with immune-mediated inflammatory diseases. This new model is integrated by physicians, pharmacists and specialised nurses and is oriented to meet the needs and expectations of the patients.

## Introduction

Immune-mediated inflammatory diseases (IMIDs) are a broad group of chronic diseases with high prevalence and diverse clinical manifestations, affecting 5–7% of the population in Europe and the United States [1]. IMIDs can cause organ damage and generating disability, have a high socio-sanitary impact and a reduction in the quality of life of patients [2,3]. The advent of biological therapies and targeted molecules has led to a remarkable change in the approach to these diseases, dramatically improving their prognosis but increasing the complexity of their pharmacotherapeutic management. In addition, approximately 20% of these patients have comorbidities and a significant group suffer from two or more coexisting IMIDs, making management handling even more challenging [4]. Studies suggest that approximately 20–30% of patients with IMIDs, such as inflammatory arthritis, inflammatory bowel disease, and psoriasis, may have multiple co-occurring autoimmune diseases [5]. For example, a patient with both rheumatoid arthritis and inflammatory bowel disease requires the coordinated care from a rheumatologist, a gastroenterologist, a pharmacist and a nurse to manage overlapping symptoms, optimise treatment, and minimise potential drug interactions.

Value-based healthcare has gained importance in managing chronic conditions, prioritizing patient-centered outcomes over service volume. This approach is especially relevant for patients with IMIDs, whose complex and multifaceted needs require a coordinated, multidisciplinary care model. Comprehensive IMIDs care models embody value-based principles by bringing together expertise across specialties, enabling early diagnosis, personalized treatment plans, and continuous monitoring of outcomes [6]. In this context, patient experience is recognised as one of the fundamental pillars of healthcare quality, alongside clinical effectiveness and patient safety [7,8,9]. Research has consistently shown that positive patient experiences correlate with improved clinical outcomes, as they are linked to enhanced treatment safety and effectiveness [10]. Enhancing patient experience through effective communication, timely access to care, and integrated health services is thus essential not only for satisfaction but also for better clinical results. This includes increasing knowledge and awareness for patients and physicians and the active participation of patients in health policy [11].

Other key strategy in the management of IMIDs is to improve communication between patients and healthcare providers [12]. Effective communication fosters a collaborative, patient-centered approach that is essential for managing IMIDs successfully. Digital health tools, including telemedicine platforms and mobile applications, are increasingly facilitating “connected care” by allowing real-time data sharing between patients and providers. These technologies enhance proactive monitoring of patient risks and support preventive care strategies [13,14,15]. By integrating different care modalities, these tools help clinicians optimise their time and resources, ultimately leading to better clinical outcomes and more efficient, value-driven care.

The cross-disciplinary nature and the associated complexity of caring for patients with IMIDs presents a challenge to the traditional healthcare system, requiring a transformation existing clinical management models. In this context, interdisciplinary teamwork is essential to provide comprehensive and cost-effective care. As a result, multidisciplinary care for patients with IMIDs is widely recommended [16,17,18,19,20,21,22,23,24]. However, specific models for implementing this approach remain scarce, and evidence of its effectiveness is limited [25,26]. We describe the design, implementation and evaluation of a new collaborative model for the integrated care of patients with IMIDs in a tertiary hospital.

## Ethical aspects

The protocol was approved by the Ethics Committee of the hospital, in accordance with the principles of the Declaration of Helsinki 2008.

## Description of the care practice

### Development and implementation

#### Setting

This project was carried out at the Hospital General Universitario Gregorio Marañón (HGUGM), a tertiary care teaching hospital of the Madrid Public Health Service (Spain). This hospital is a reference in terms of complexity for the entire Community of Madrid for various specialities or complex procedures, which increases its referenced population to more than 750,000 people.

The HGUGM has a long history of caring for patients with IMIDs following the traditional model of clinical care. This consists of rheumatology, gastroenterology and dermatology clinical services, which mainly treat patients with the pathologies of rheumatoid arthritis, spondyloarthritis, psoriatic arthritis, inflammatory bowel disease and psoriasis, respectively, as well as other pathologies with less prevalence. These clinical services are independent in both clinical and logistical management, with different service managers, are physically located in different parts of the hospital and report directly to the hospital management team. Patients with each pathology are treated in the appropriate service. Patients with co-morbidities who needed to be treated by more than one service had to manage their appointments independently in the different locations. In this model, patients had to attend different appointments on different days and in different locations in order to by seen the appropriate professionals.

#### Stages of implementation

In 2018, a multidisciplinary team leader from the departments of rheumatology, gastroenterology, dermatology and pharmacy was created to design and implement an innovative health model for the comprehensive care of patients with IMIDs. This new healthcare model was called CEIMI (Centre for the Care of Patients with IMIDs).

The first stage of development was to identify the weaknesses of the traditional model and prioritise solutions. To this end, multi-professional brainstorming sessions were convened, based on a cause-effect analysis of the initial situation. This was complemented by a qualitative research study using focus groups to learn about patients’ experiences of the disease process and the social and health care they receive. Finally, a consulting firm was engaged to assist with the resource assessment. Table 1 shows the main shortcomings identified and the proposed solutions.

**Table 1:** Challenges, barriers and proposed solutions to the new model of care.


CHALLENGE	BARRIER	SOLUTION

***Direction and Strategy***		

Several clinical services involved without common governance.	Conflict of powers between heads of service.	Transversal leadership system. Semi-annual rotation of the leadership position.

Incorporate patients into decision making.	Absence of references in the institution.	Incorporation of patients into the governance team.

The best multidisciplinary model of care and preferential circuits is not defined.	Absence of references.	Creation of a committee of experts. Bibliographic review. Hiring a specialized consulting firm.

Unify the center in the same location.	Need to renew facilities. New location available away from the general hospital.	Search for financing for the works. Transfer between locations.

Ability to respond to expected patient demand.	High care volume workload. Call effect due to the center’s novelty.	Prioritize patients treated with biological therapies or targeted molecules.

***Processes Management***		

Patient-centered clinical care.	System focused on the process.	Redefinition of processes. Preparation of a patient-centered process map.

Patients with more than one IMIDs not correctly integrated.	Complexity in the management of these patients by different professionals.	Creation of a multidisciplinary committee for the management of these patients.

Professional maintenance of each patient.	Center far from individual services.	Reconciliation of the agendas of the professionals involved.

Incorrect referral of patients.	Lack of knowledge by professionals.	Dissemination plan for the criteria for referral to the center.

Continuous evaluation of the results.	Difficulty of data exploitation.	Development of a specific dashboard. Collaboration with the hospital’s quality service.

***People***		

Need for additional personnel to cover the service.	Lack of resources to hire new personnel.	Conciliation and displacement of previously available personnel.

***Alliances***		

Improve communication with patient.	Lack of resources.	Search for financing to develop telemedicine projects.

Improving continuity of care between different levels.	Poor coordination with primary care.	Preparation of a training and communication plan between care levels.

National and international recognition as a reference center.	–	Seek support from hospital management and industry. Preparation of an image and dissemination plan.


**Figure 1:**
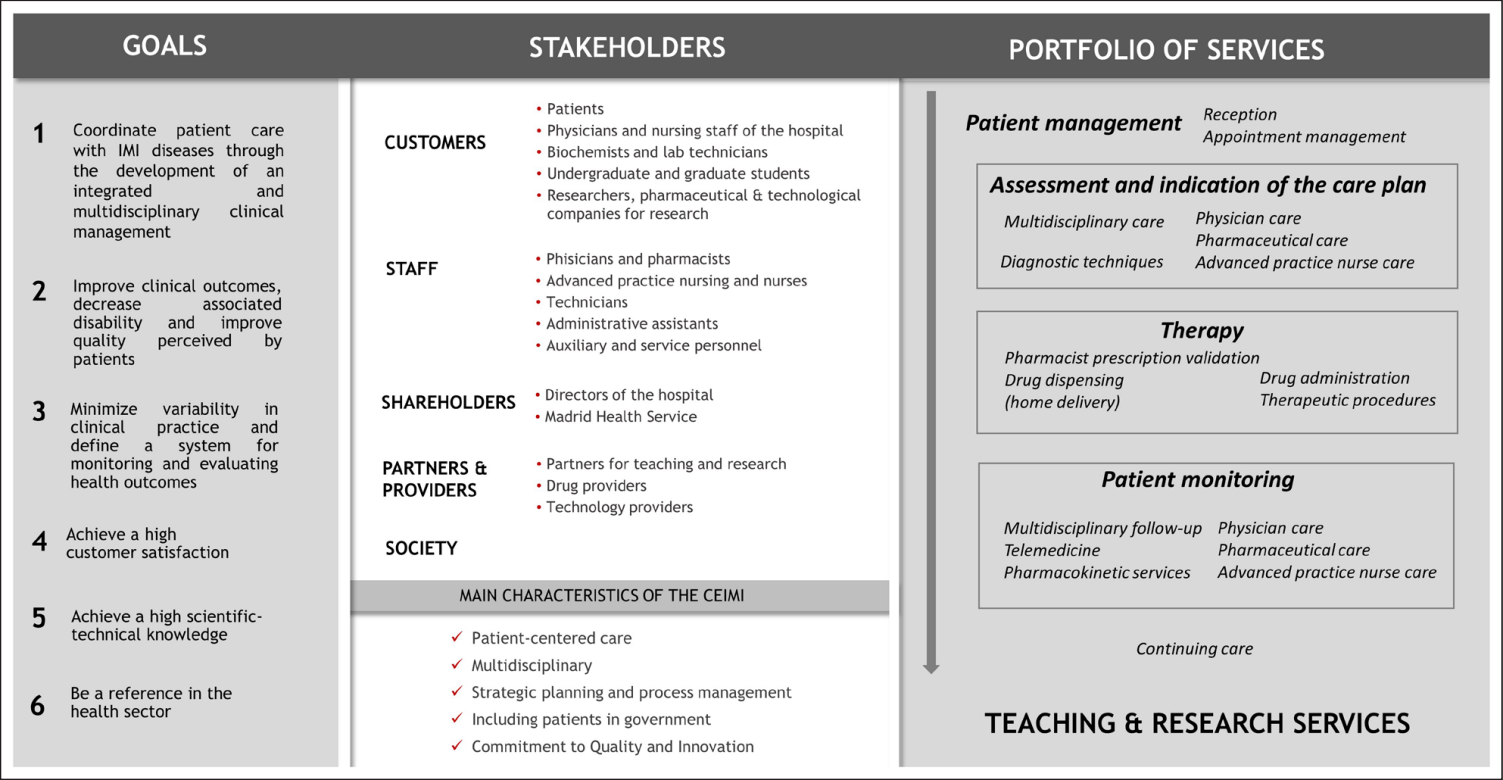
Goals, stakeholders and portfolio of services of the CEIMI.

In order to respond to all these needs, a multidisciplinary and highly specialised centre was designed, functionally and hierarchically integrated into the HGUGM. CEIMI defined its main objectives, its stakeholders and its service portfolio (Figure 1). The main initiatives implemented are detailed in Table 2.

**Table 2:** Main initiatives implemented.


***Direction and Strategy***

Definition of the CEIMI’s mission, vision, and values

Definition a transversal leadership system, in cooperation with all the hospital services involved: establishment a Steering Committee to improve communication and facilitate decision-making

Incorporation of the patient into the governance system. We use the satisfaction surveys to evaluate patient experienced.

Strategic planning deployed to annual operational plans, with short-term objectives and assignment of responsibilities

Implementation of a scorecard and annual SWOT analysis according to stakeholders’ needs and CEIMI performance

Creation a Case Committee to make a collegiate and shared decision about the most appropriate care plan for each patient

Creation a multidisciplinary consultation in which patients with different concomitant IMIDs pathologies were attended by different specialists simultaneously

Carrying out focus groups for humanization where the needs and expectations of patients, doctors, nurses and service personnel have been collected

Creation of the APN role with a key role in the coordination of care activity, in the planning, management of care and treatment

Implementation of a program for the individualization of pharmacotherapy (Precision Medicine) to monitoring drug and antibody levels and to evaluate polymorphisms with the potential to predict their response or toxicity

Implementation of a tele-assistance service for ensure continuous patient care

***Processes Management***

Design of an integrated and coordinated patient care circuit Definition of the process map of the CEIMI, assignment of the person responsible for each process and supervision through the Process Control Card, with monitoring indicators

Establishment of operational management meetings order to improve internal management

Implementation of a custom scorecard with short- and long-term indicators covering patient, customer and people satisfaction, society, activity and health outcomes

Definition of a structured calendar for the evaluation of different health outcomes to all professionals

Incorporation of Patient Reported Outcomes (PRO) into clinical practice including quality of life, health status, symptoms present, adherence and satisfaction. These PRO are measured through rigorously developed and validated questionnaires

Design, use, and analysis customer surveys to optimize CEIMI processes.

***People***

Creation of a welcome plan for staff

Definition of an organizational manual, that contains the organizational and functional description of the same, as well as the definition of the competency profiles of each of the professionals

Design, use, and analysis of work climate surveys to evaluate staff motivation and satisfaction, including their participation in the leadership system, regularly and to compare data with those of other organizations

Implementation of individualized development plans and structured training plans for all staff

***Alliances***

Improvement in partnership management, with objectives, monitoring and improvement actions defined for each alliance

Implementation of surveys for partners

Design and implement a School of Patient to reinforcing the education of the patient and their families

Developed training videos for patients in collaboration with Madrid School of Public Health

Systematization of innovation management and implementation of information systems technologies in all phases of the drug use process: i. CPOE in all consultations. ii. Advanced CDS system to support pharmaceutical validation; iii. e-MAR in Day Hospital; iv. Barcode control for treatments administered at Day Hospital; v. Robotization of dispensing in the Outpatient Pharmacy; vi. Home follow-up of patients using an in-house development App; vii. Home delivery system to prevent the most vulnerable patients from traveling to the hospital.


##### Leadership and Management structure

**Figure 2:**
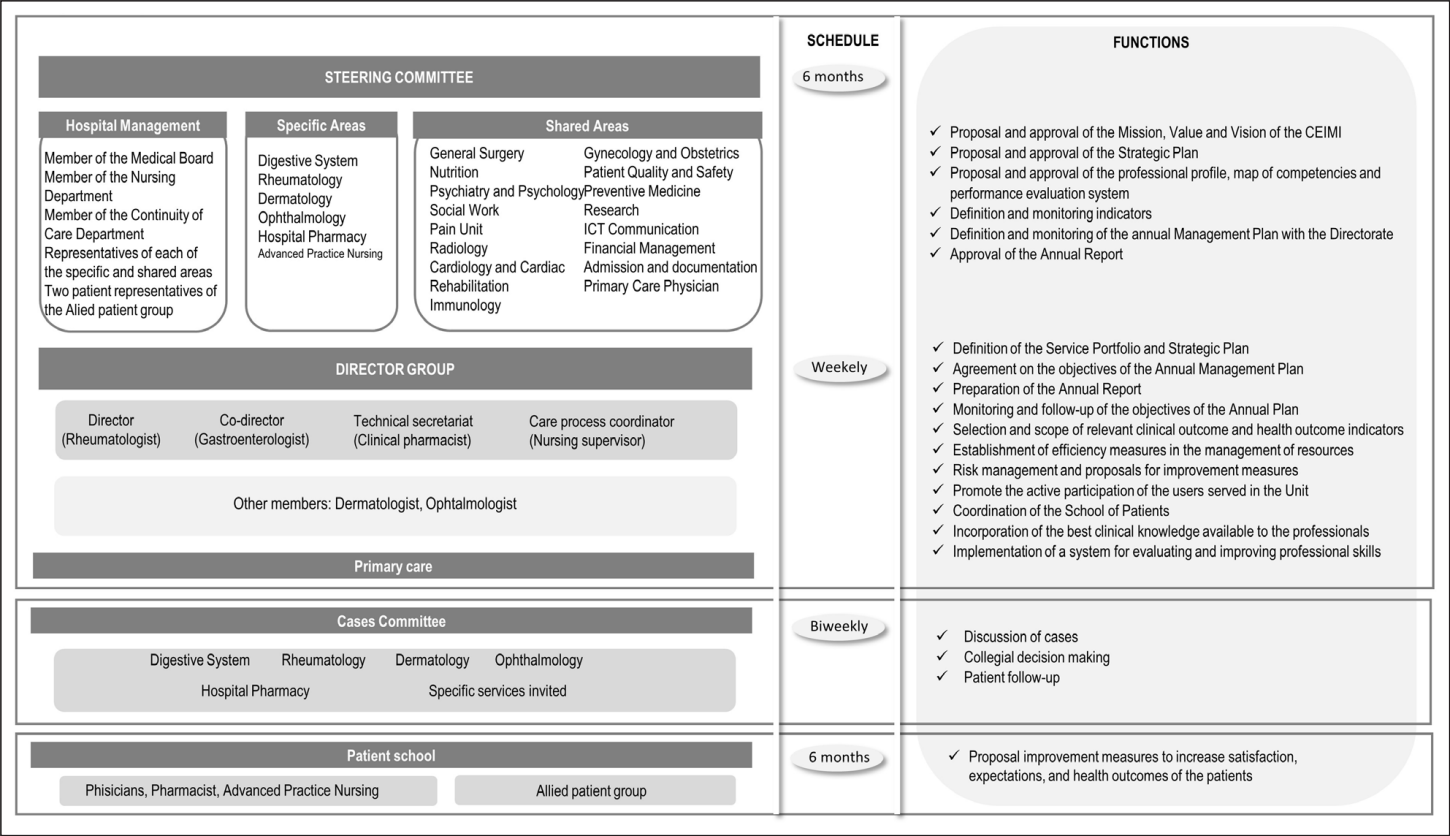
Management structure.

The CEIMI is managed by a transversal governance system in collaboration with all the hospital services involved, including the hospital medical directorate (Figure 2). It includes a directorate formed by a multidisciplinary team that reports directly to the hospital’s medical directorate. It is responsible for the operational handling to facilitate the clinical management of the CEIMI. It is part of the Steering Committee and the position of Director rotates every 6 months.

The Steering Committee includes members from CEIMI’s specific areas of expertise, two patient representatives and general practitioners. It is responsible for setting the strategic framework and ensuring its understanding throughout the CEIMI, facilitating decision making, involving all staff in short term action plans and evaluating the performance of the CEIMI on a quarterly basis. The Cases Committee has been set up to discuss specific patients with complex management, to make a collegial and shared decision about their care plan and to monitor them.

Ten expert patients with IMIDs form the allied patient group. The aim was to enable active patient participation in the governance system. The focus group technique is used to elicit patients’ experiences of the care process for their condition, as well as what needs to be improved to increase their satisfaction, expectations and health outcomes. In addition, two representatives of the allied patient group are included in the CEIMI Steering Committee. The specific functions of each component of the governance structure are detailed in Figure 2.

##### Organizational structure and reorganization of processes

CEIM is undertaking a strategic positioning exercise to align with the overall global strategy of HGUGM and update it for the new environment. CEIMI’s strategic objectives are described in Figure 1. The prioritisation of objectives by the CEIMI Steering Committee included the standardisation of all processes and their certification. In 2020, CEIMI obtained the UNE-UE-ISO: 9001-2015 certification. This implies compliance with high standards in the design, monitoring and improvement of processes. A dashboard of process result indicators has been prepared.

CEIMI’s management system is based on Management by Processes to integrate the different norms, models, standards and requirements in order to provide maximum quality and safety for patients. The following references have been considered and integrated into the strategy EFQM model, ISO 900 standards, Joint Commission International or ISO 14001 standard.

##### Optimization and protocolization of health care practice

One of the main goals of CEIMI was to minimise variability in clinical practice in order to improve clinical outcomes for patients. All clinical processes involving doctors, pharmacists and nurses were recorded. We also defined a structured calendar for the assessment of different health outcomes. Clinical guidelines were developed, including diagnostic and treatment algorithms and clinical monitoring. The guidelines also included the analysis of predictive factors of drug response, tapering strategy in patients in complete remission and therapeutic strategy in patients with poor response. A special effort was made to optimise the early diagnosis of other IMIDs.

##### Patient care circuit- Coordination of Assistance Activity

Patients were cared for in a multidisciplinary way, following an agile and integrated/coordinated workflow adapted to the different IMID conditions. In this way, the entire care process for patients with IMIDs was carried out on the same day and in the same place to avoid unnecessary hospital visits. In addition, CEIMI had its own administrative processes: a one-stop office was set up to provide patients with all the necessary appointments. An integrated appointment system with management software was implemented. This made the patient’s itinerary more user-friendly.

After reception and identification using three different identification codes, patients received a comprehensive assessment by the doctor, who indicated the initial care plan. The pharmacist and nurses then completed the clinical care of the patients. Finally, patients could leave the CEMI (in case of oral or subcutaneous treatment) or receive their intravenous therapy at the infusion centre. In addition, internal alliances were established with other services (psychiatry, nutrition) involved in the care of these patients and with primary care services to ensure continuity of care.

The renovation of the facilities was necessary to change the previous structure from the traditional process-oriented model to a patient-oriented model. All professionals, whether clinical, general or ancillary, were able to have a space for their activities, allowing the implementation of new multidisciplinary and integrated work circuits. The CEIMI includes a reception area, two waiting rooms, an infusion centre with 13 chairs, 8 multi-purpose consulting rooms and a pharmacy service with 2 pharmaceutical care consulting rooms. A meeting room and a multi-purpose room have been created, and a dedicated space (two offices) has been allocated for clinical trials, where doctors, pharmacists, nurses and trial coordinators can work together.

##### People

In order to meet the estimated health care needs, given that the new model includes an individualised care plan for patients with doctors, pharmacists and nurses, a multidisciplinary team of 24 professionals was initially recruited, including 10 doctors, 4 pharmacists, 5 nurses, 3 nursing technicians and 2 administrative technicians.

Human resources management is an essential element for the effective operation of CEIMI and aims to have qualified staff committed to the CEIMI project. Maintaining and improving quality is based on interdisciplinary work, respect for professional ethics, the commitment and participation of all staff and the use of their knowledge and creative skills. The professional development of CEIMI staff is systematically planned. CEIMI’s management continuously analyses the needs and expectations of the professionals through an annual work climate survey. In terms of training, individual development plans have been drawn up, with an annual assessment of skills. With the information gathered, objectives and actions are defined and implemented, which serve as the main axis for decision making and areas for improvement. Some of the aspects developed for CEIMI professionals to carry out a service of technical and human excellence, improving their comfort and impact are described in Table 2. These projects will make it possible to attract talent and maintain leadership.

##### Alliances

The most important initiative in terms of alliances was the creation of the School of Patients. The CEIMI School of Patients was developed as an innovative project for the humanisation of healthcare, with the aim of improving the relationship between CEIMI, the patient and their environment, and responding to their new needs in a dynamic and accessible way. The aim of the School of Patients is to improve the self-care and quality of life of people with IMIDs through a teaching and learning process. The School of Patients develops strategies aimed at promoting the development of personal health, autonomy and self-care skills to facilitate appropriate management of disease symptoms, optimal adherence to treatment and the development of healthy lifestyles and habits. These interventions include information/education, family/social support, group dynamics and behavioural reinforcement. Other initiatives include: a) a digital platform, consisting of a mobile application (app) that allows continuous two-way communication between patients and their healthcare professional and provides them with all the digital tools to promote self-care: e-MidCare® [27] (sponsored by Novartis); and b) a home delivery system to bring treatment to the homes of the most vulnerable patients so that they do not need to go to hospital, especially during the pandemic.

On 15 January 2019, the CEIMI support activities started. Table 3 shows the differences between the previous and new models of care.

**Table 3:** Differences between the old and new care model.


TRADITIONAL CARE MODEL	NEW INTEGRATED CARE MODEL	IMPROVEMENT

***Direction and Strategy***		

Absence of mutual leadership team. Address dependent on each Clinical Service.	Transversal leadership system	Collaborative government. Participation of all professionals involved in decision making.

Case Committee no available	Case Committee	Collegiate and shared decision about the most appropriate care plan for each patient

Leadership system without patients	Patients included in the leadership system	Promote responsibility and empowerment of patients

Facilities distributed in multiple locations	Center located in a single location	The whole care process was performed on the same day and in the same place to avoid unnecessary hospital visits

Patients with more than one IMIDs	Mutidisciplinary consultations	Patients with different concomitant IMIDs were attended simultaneously in the same room by two or more different specialists

Model without nurses or with non-specialized nurses	Incorporation the figure of the APN with a key role	APN provide personalized and comprehensive quality care to patients and their environment through a care model of shared decision, patient-centered and coordinated between the different levels of care and health professionals

***Processes Management***		

Process-centered model	Patient-centered model	Improving quality of care and patient experience

Multiple clinical circuits not coordinated with each other	Integrated and coordinated patient care circuit	Optimization of healthcare resources and improving patient experience

Evaluation plan not defined and dependent on each service	Implementation an evaluation plan	Measure results and optimize objectives

No standardized measurement of results or evaluation of processes	Implementation of a custom scorecard covering all process	Facilitates analysis and peer comparison

Patient Reported Outcomes not available	Incorporated Patient Reported Outcomes into clinical practice	Improvement clinical care

***People***		

The competencies of each professional are not adequately defined	Development an organizational manual with adequate definition of competencies	Optimization of personal resources and improving professionals satisfaction

Welcome plan for staff not available	Creation of a welcome plan for staff	Improved staff satisfaction

***Alliances***		

E-health tools not available	Teleconsultation and app available	Communication and real-time monitoring of patients and provide additional data to support clinical decision-making, improve the quality of care, and contribute to the empowerment of patients

Poor coordination with others services and primary care	Creation of internal alliances with different services and primary care	Improved coordination between care services and levelsnand the continuity of health care

School of Patient not available	Implementation of a School of Patient	Improve the education of the patient and their families


### Evaluation

#### CEIMI bases its management structure on three basic tiered mechanisms

##### Direction and Strategy

A multi-year strategic planning process is carried out, with an annual review, in which the trends of stakeholder, the environment and performance results (scorecard, customer and employee results reports, process results, etc.) are analysed.

The strategy is applied to annual plans structured by key processes and projects, with objectives for this horizon that are aligned with the objectives of the Hospital and the Madrid Health Service.

The annual plan is drawn up and periodically monitored by the Steering Committee, which is made up of a representative of the HGUGM management team from the medical area, nursing and continuity of care areas, as well as representatives from each of the specific knowledge areas and from the shared knowledge areas (support areas).

Some of the initiatives developed were; firstly, the Case Committee was created. Second, a multidisciplinary consultation model was implemented, where patients with different concomitant IMID were seen simultaneously in the same room by two or more different specialists. Thirdly, in order to avoid additional trasnfers of patients outside the CEIMI, ophthalmology, nutrition, and preventive medicine were incorporated into the CEIMI due to the high prevalence of this type of comorbidity.

##### Process Management

Those responsible for the processes structure them, monitor their progress throughout the year and carry out the annual “process control sheet”, in which they analyse their results qualitatively and quantitatively and define areas for improvement and objectives for the following year. For example, the implementation of the continuous survey has led to some projects to optimise CEIMI’s processes. Some examples of key performance indicators are shown in Table 4.

**Table 4:** Key performance indicator.


CLASS	INDICATOR	PERIODICITY OF EVALUATION

***Activity***	Nº of attended patients	Quarterly

	Nº of patients treated with BT or TT	Quarterly

	Nº of new patients starting treatment at CEIMI	Quarterly

	Nº of physician’s consultations	Quarterly

	- Nº of telematic consultations	- Quarterly

	- % of unscheduled consultations	- Quarterly

	Nº of pharmacist consultations	Quarterly

	- Nº of telematic consultations	- Quarterly

	Nº of nurse consultations	Quarterly

	- Nº of telematic consultations	- Quarterly

	Nº of drug dispensations	Quarterly

	Nº of home delivery dispensations	Quarterly

	Nº of sessions in Day Hospital	Quarterly

	Nº of patients attended at Day Hospital	Quarterly

	% patients treated of the Health Area	Quarterly

***Health***	Age, mean (SD)	Annual

	% Male	Annual

	Effectiveness	

	% of patients in remission*	Annual

	% of patients admitted to hospital	Annual

	Nº hospital admission/patient/year	Annual

	Average stay of admission/patient	Annual

	% of patients with emergency room visits	Annual

	Nº emergency room visits/patient/year	Annual

	Safety	

	% of patients with treatment suspension due to toxicity	Quarterly

	Severe infusion reaction	Quarterly

	Nº of patients with severe extravasation problems	Quarterly

	Quality of live	

	EUROQOL, median (IQR)	Annual

	HAD, median (IQR)	Annual

	WPAI**, %	Annual

	WPAI***, mean (SD)	Annual

	QOL-RA, median (IQR)	Annual

	AsQOL, median (IQR)	Annual

	PsAQOL, median (IQR)	Annual

	IBDQ, median (IQR)	Annual

	DLQUI, median (IQR)	Annual

	Efficiency	

	% of patients with tapering of the treatment	Quarterly

	% of patients with intensification of treatment	Quarterly

	% of patients with biosimilar molecule****	Quarterly

	Average cost per patient/year	Quarterly

	% patients with lower cost counseling	Quarterly

	Quality	

	% of patients with adherence > 90%	Annual

	% of patients with drug levels	Quarterly

	% of patients with antibodies anti-drug	Quarterly

***Satisfaction***	Patient	

	Average score on outpatient satisfaction	Annual

	With physicians	Annual

	With pharmacist	Annual

	With APN	Annual

	Coordination between professionals	Annual

	Customer	

	Average score on internal customer satisfaction	Biannual

	Average score on external customer satisfaction	Biannual

	Average score on student/resident satisfaction	Biannual

	People	

	Average global score on the work climate survey	Biannual

	Score of Physicians/pharmacist/nurses	Biannual

	Score of other non-sanitary staff	Biannual

	Score in specific aspects	Biannual

***Society***	Nº of clinical trials	Annual

	Nº of research projects	Annual

	Nº of publications	Annual

	Annual impact factor of scientific publications	Annual

	Nº of courses	Annual

	Nº of conferences	Annual

	Nº of visits received by other organizations	Annual

	Nº of mentions in the media	Annual

	Nº of twitter followers	Annual

	Nº of audiovisual tools	Annual

	Nº of prizes awarded	Annual


##### Operational Management

During the first stage of development to identify the weaknesses of the traditional model and prioritise solution, we found the following deficiencies: the best multidisciplinary model of care and preferential circuits is not defined (prioritised by patients), patients with more than one IMID are not correctly integrated in our health system (prioritised by patients and professionals), the high care volume workload due to the incidence increase in chronic diseases and the high economic impact on health system and society (prioritised by professionals).

The area managers, together with their key personnel, meet periodically (weekly, fortnightly, monthly – depending on the characteristics of their activity) to analyse the problems and to present and explain the documentation of the Quality Management System (manual, procedures, instructions, etc.).

The continuous evaluation and the results of the surveys, evaluated by the Steering Committee, made it possible to identify unmet needs, which were included in the SWOT (Strengths, Weaknesses, Opportunities, and Threats) and led to several improvements in the care new model:

- Adding a member from dermatology and ophthalmology to the Director Group.
- Extension of consultation days for specialities with a higher care load.
- Inclusion of a diagnostic ultrasound consultation in the service portfolio.
- Implementation and consolidation of the School of Patients to strengthen the education of patient and their families.
- Review and update of quality of life questionnaires. Inclusion of a questionnaire to assess insomnia.
- A CEIMI website with the requested information has been developed as an improvement derived from the satisfaction surveys of internal and external partners.

Other examples of improvements derived from patient satisfaction surveys: the creation of a complaints and acknowledgement circuit, the modification of the waiting room furniture, the increase in the number of chairs in the waiting rooms and screens for the appointment system, the implementation of a queue management system to reduce waiting times and to guarantee confidentiality, the consolidation of the telematic consultation and home dispensing by the pharmacy service, and consolidation and expansion of de features of the App.

## Discussion

This project presents the development, implementation, and evaluation of the pioneering CEIMI health care center, designed to offer comprehensive, multidisciplinary care for patients with IMIDs treated with biological therapies and targeted molecules. CEIMI represents a transformative shift from traditional care models, introducing a cross-disciplinary governance structure that actively integrates patient participation to meet patient-centered care needs and expectations.

Although multidisciplinary care for IMIDs patients is broadly recommended [16,17,18,19,20,21,22,23,24], there are limited guidelines tailored to IMID-specific models, and the few studies available offer mixed findings on their efficacy [15,16]. Some researchers have proposed optimal management guidelines for IMIDs through a multidisciplinary approach, though often without specific program outcomes [18,21,28]. These recommendations emphasize the integration of IMID units within institutional frameworks and advocate for the inclusion of a diverse team of dermatologists, gastroenterologists, rheumatologists, specialized pharmacists, nursing staff, and administrative support. Additionally, IMIDs units are expected to provide not only clinical services but also contribute to teaching and research, ensure measurement of health outcomes, adhere to agreed-upon protocols, and maintain dedicated administrative processes [4]. To our knowledge, CEIMI is the first center to fully implement these criteria, advancing IMID care by focusing on the early diagnosis and management of concurrent IMIDs, which account for 12.6% of the patient population. To optimize the care of these patients, CEIMI has established a Case Committee for shared decision-making on individualized treatment plans, and provides simultaneous multidisciplinary consultations.

A significant innovation within CEIMI’s organizational framework is the transformation of traditional governance models into an inclusive and collaborative health care structure. Efforts were focused on creating a cross-functional governance team that integrates input from all relevant hospital services and incorporates patient voices in health policy decision-making. This cohesive, collaborative model facilitates improved continuity and accessibility of care for IMID patients, enabling streamlined communication among professionals, increased adherence to evidence-based practices, and a reduction in patient hospitalization duration. Studies confirm that collaborative care models enhance decision-making, promote innovation, boost compliance with medication protocols, and improve both quality audit outcomes and patient psychosocial support [29,30,31,32,33]. Furthermore, employee satisfaction and retention rates are higher in organizations where teamwork and a culture of quality and safety are prioritized [34]. Despite these benefits, the literature also highlights challenges associated with collaborative care, including varying routines, expertise, professional identities, hierarchical dynamics, and time limitations [35]. To address these challenges, CEIMI conducts an annual work climate survey, incorporating feedback from all participating professionals into a structured SWOT analysis, which serves as a core decision-making tool to drive continuous improvement.

In response to the growing focus on value-based healthcare, CEIMI adopts a patient-centric approach to healthcare policy and management. This model underscores the importance of transformational leadership in health management, while emphasizing proactive engagement of patients and the community across all aspects of healthcare delivery [36]. By including patients in Clinical Management, CEIMI fosters patient accountability and empowerment, which contributes to better health outcomes and reduced healthcare costs [29,37,38,39]. Patient involvement in healthcare governance, however, remains limited across most systems. CEIMI’s model addresses this gap by actively involving patients in governance roles and decision-making processes. The center also uses focus groups to address the humanization of care, gathering valuable insights on patient needs and expectations. Additionally, CEIMI has established a Patient School to promote self-care skills and knowledge acquisition, aiming to improve patient outcomes and quality of life as supported by previous studies [12,29].

CEIMI also emphasizes the importance of standardized health outcome monitoring, enabling the analysis, comparison, and sharing of knowledge regarding IMIDs management. Furthermore, the center is dedicated to continuous education for both patients and healthcare providers and to fostering high-quality research that attracts talent and strengthens partnerships with national and international institutions. CEIMI’s comprehensive approach has yielded promising results, improving both care efficiency and patient quality of life, with high levels of satisfaction reported across stakeholders. The publication of these results is forthcoming, marking an important contribution to the broader field of IMID management.

### Limitations

This project has a limitation due to its specific characteristics. It was carried out in a relatively large public hospital, with a wide range of internal and external clients, and with considerable experience in process improvement and technology implementation, which significantly influenced its development. Consequently, these results could not be obtained in centres with different characteristics. Nevertheless, these results could help other hospitals to implement a similar model, since this is one of the longest reported experiences and the first experience in a centre designed exclusively for the treatment of IMID patients.

## Conclusion

CEIMI is a new collaborative care model for patients with immune-mediated inflammatory diseases, integrating specialists in rheumatology, gastroenterology, dermatology, ophthalmology, endocrinology and nutrition, preventive medicine, psychiatry, pharmacist and advanced practice nurses, working with the common goal of providing patients with comprehensive and interdisciplinary care that is tailored to their needs and expectations. It is governed by a transversal leadership system in cooperation with all the hospital services involved, including the Medical Directorate of the hospital and the patients, promoting greater responsibility and empowerment. This model facilitates the coordination of all professionals, the protocolization and systematization of clinical care to patients, and the improvement of follow-up and monitoring of health outcomes through the definition of indicators.
